# OP05 Efficiency Frontier Analysis Of Ciltacabtagene Autoleucel For Relapsed/Refractory Multiple Myeloma In Brazil

**DOI:** 10.1017/S0266462324000692

**Published:** 2025-01-07

**Authors:** Marcus Carvalho Borin, Ludmila Gargano Álex Brunno do Nascimento Martins, Bárbara Rodrigues Alvernaz dos Santos, Isabela Freitas, Francisco de Assis Acurcio, Juliana Alvares-Teodoro, Augusto Guerra

## Abstract

**Introduction:**

Multiple myeloma (MM) is a challenging hematological malignancy, primarily treated with autologous stem cell transplantation (ASCT). However, relapse or refractoriness is inevitable, necessitating alternative treatments. This study evaluates ciltacabtagene autoleucel (Carvykti®), a novel therapy, against a second ASCT, using an efficiency frontier approach to assess its therapeutic value and cost-effectiveness.

**Methods:**

We conducted a comparative analysis using data from CARTITUDE-1 clinical trials and a Brazilian real-world cohort (2002 to 2015) of MM patients treated under SUS (Brazilian Healthcare System). We estimated survival curves and area under the curve (AUC) for both interventions over 48 months and projected the curves for a 10-year horizon using parametric distributions. Cost-effectiveness was assessed by calculating the incremental cost per month of survival. Efficiency frontier methodology was employed to determine a proportional price for ciltacabtagene autoleucel, based on the cost and median survival benefits compared to the second ASCT.

**Results:**

Ciltacabtagene autoleucel demonstrated a 7.27 percent increase in AUC for overall survival over 48 months compared to the second ASCT. The incremental cost was BRL54,219.15 (USD11,133.30) per month of survival. Over a 10-year horizon, the estimated cost for ciltacabtagene autoleucel was significantly higher than that for the second ASCT. Using the efficiency frontier approach, the cost of ciltacabtagene autoleucel should not exceed BRL228,226.42 (USD46,863.74), considering its survival benefit and cost of production.

**Conclusions:**

Ciltacabtagene autoleucel demonstrates significant anti-tumor activity in relapsed/refractory MM, with a notable survival advantage. Efficiency frontier analysis suggests a maximum justified cost, providing a framework for pricing decisions. This study highlights the importance of balancing innovation with cost-effectiveness in healthcare decision-making.

